# Role of NLRP3 inflammasome in central nervous system diseases

**DOI:** 10.1186/s13578-024-01256-y

**Published:** 2024-06-07

**Authors:** Lu Zhang, Yufen Tang, Peng Huang, Senlin Luo, Zhou She, Hong Peng, Yuqiong Chen, Jinwen Luo, Wangxin Duan, Jie Xiong, Lingjuan Liu, Liqun Liu

**Affiliations:** 1 Department of Pediatrics, The Second Xiangya Hospital of Central South University, Changsha, 410011 China; 2 Department of Pediatric Neurology, Children’s Medical Center, The Second Xiangya Hospital of Central South University, Changsha, HuChina 410011 China

**Keywords:** NLRP3, Inflammasome, Central nervous system, Immune disease, Biological function, Inhibitors

## Abstract

The central nervous system (CNS) is the most delicate system in human body, with the most complex structure and function. It is vulnerable to trauma, infection, neurodegeneration and autoimmune diseases, and activates the immune system. An appropriate inflammatory response contributes to defence against invading microbes, whereas an excessive inflammatory response can aggravate tissue damage. The NLRP3 inflammasome was the first one studied in the brain. Once primed and activated, it completes the assembly of inflammasome (sensor NLRP3, adaptor ASC, and effector caspase-1), leading to caspase-1 activation and increased release of downstream inflammatory cytokines, as well as to pyroptosis. Cumulative studies have confirmed that NLRP3 plays an important role in regulating innate immunity and autoimmune diseases, and its inhibitors have shown good efficacy in animal models of various inflammatory diseases. In this review, we will briefly discuss the biological characteristics of NLRP3 inflammasome, summarize the recent advances and clinical impact of the NLRP3 inflammasome in infectious, inflammatory, immune, degenerative, genetic, and vascular diseases of CNS, and discuss the potential and challenges of NLRP3 as a therapeutic target for CNS diseases.

NLRP3 is a protein consisting of an amino-terminal Pyrin domain (PYD), a central Nacht domain, and a C-terminal leucine-rich-repeats domain (LRR domain). Once primed and activated, it can complete the assembly of inflammasome (sensor NLRP3, adaptor ASC, and effector Caspase1), resulting in caspase-1 activation, IL-1β and IL-18 release enzyme cleavage and activity increase, as well as to pyroptosis, thus playing an important role in regulating innate immunity and autoimmune diseases [1]. In contrast to the attention paid to autoimmune and infectious diseases of the CNS, the role of immune mechanisms in degenerative and epilepsy and other CNS diseases has long been neglected. In recent years, the booming experiment and inspection technology have provided a large number of evidences for immune mechanisms are involved in CNS diseases, including but not limited to autoimmune and infectious diseases, and have shown potential therapeutic effects in modulating immune signaling mechanisms. The NLRP3 inflammasome was the first one studied in the brain, with impressive results. Numerous studies have demonstrated that the NLRP3 inhibitor MCC950, the cystic fibrosis transmembrane conductance regulator inhibitor C172 and its analogs CY-09,and oridonin show good curative effect in various preclinical immunopathological models such as Alzheimer’s disease, traumatic brain injury, atherosclerosis, diabetic encephalopathy, human immunodeficiency virus (HIV)-associated neurocognitive disorders (HANDs), cerebral edema, cerebral ischemia-reperfusion injury, hypoxic ischemic encephalopathy, etc [1–6]. The application of NLRP3 inflammasome inhibitors in CNS diseases will be eagerly awaited. In this review, we summarize the latest biological characteristics of NLRP3 inflammasome, how the inflammasome is primed and activated in CNS infectious, inflammatory immune, degenerative, vascular, genetic, metabolic, tumor and epilepsy diseases and its role in the occurrence and development of diseases after activation, explore the potential and challenges of NLRP3 as a therapeutic target for CNS diseases.

##  Overview of NLRP3 inflammasome

###  NLRP3 inflammasome priming and activation

**Fig. 1:**
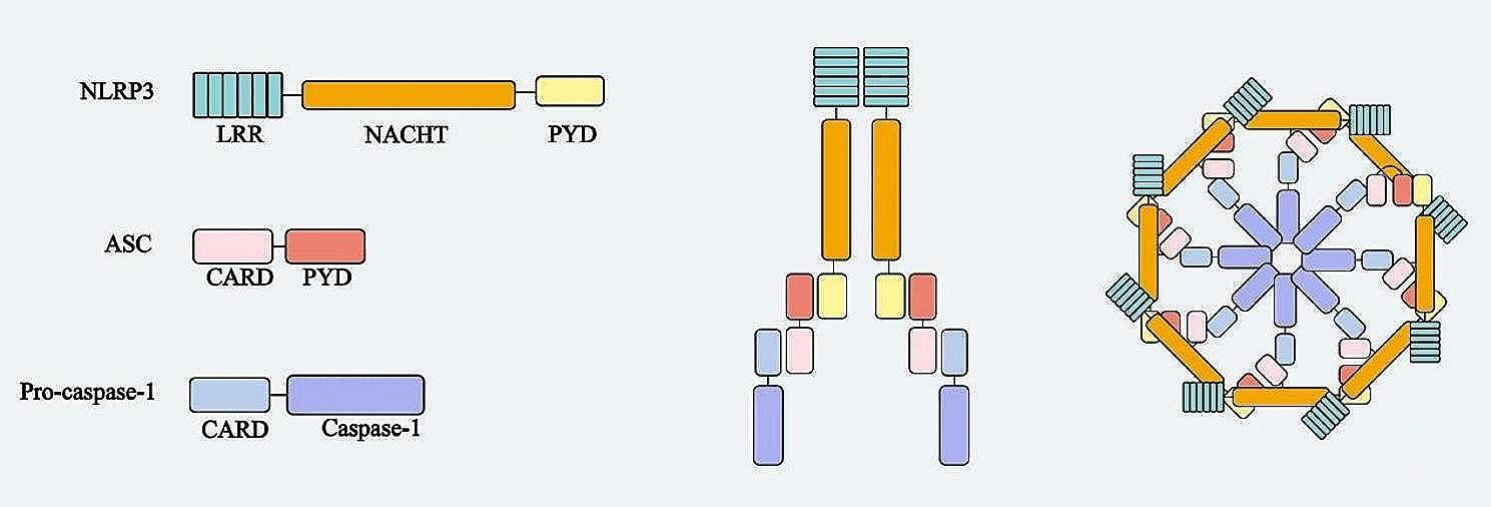
Schematic representation of NLRP3 inflammasome structure.NLRP3 inflammasome, consisting of the sensor (NLRP3), adapter (ASC) and effector (caspase-1)

**Fig. 2:**
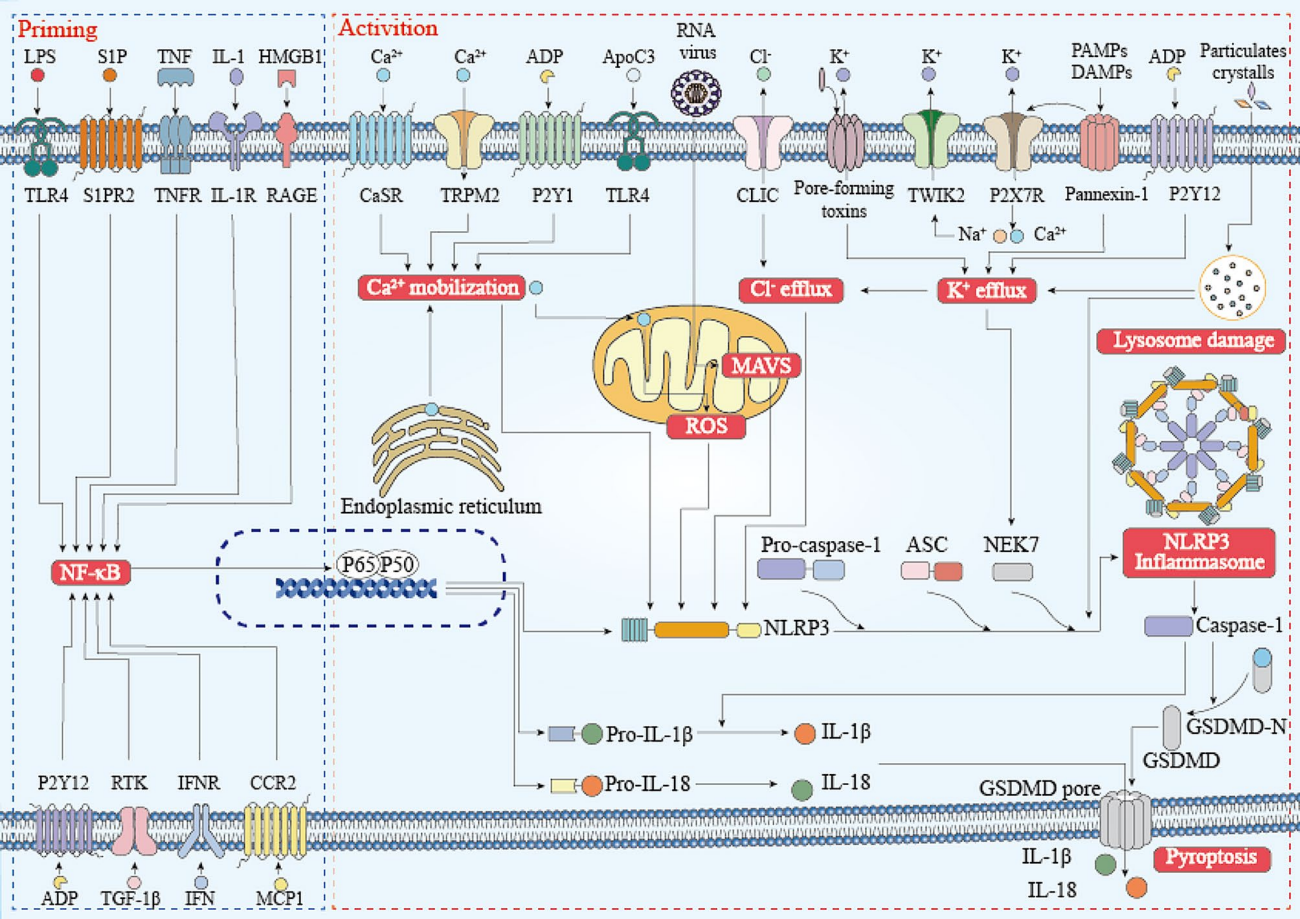
Canonical activation of the NLRP3 inflammasome pathways. NLRP3 canonical activation requires two steps, a priming step and an activation step.The priming is achieved through the identification of pathogen-associated molecular patterns and damage-associated molecular patterns. TNF, IL-1, HMGB1, IFN, TGF-1β and LPS could upregulate NLRP3 expression by inducing nuclear NF-κB activation.Primed NLRP3 induces inflammasome assembly and full activation upon recognition of K + efflux, Cl − efflux, Ca2 + mobilization, lysosome destruction, anti-Golgi dissociation, mitochondrial dysfunction, mitochondrial ROS production and mitochondrial DNA release into the cytosol.NLRP3 inflammasome formation includes binding of NLRP3 with NEK7, oligomerization of NLRP3, assembly of ASCs into fibrils, and recruitment and activation of Caspase 1. Actived NLRP3 inflammasome can induce caspase-1-dependent pyroptosis in host cells. Active GSDMD, which bind to phosphatidylinositol phosphates and phosphatidyl serine in the membrane leaflets, aggregate and insert into the plasma membrane to form a pore. Inflammatory cytokines such as IL-1β and IL-18 are released into the interstitial space and activate corresponding receptors in adjacent cells, causing an immune cascade and amplifying the injury effect

NLRP3 inflammasome, consisting of the sensor (NLRP3), adapter (ASC; also known as PYCARD) and effector (caspase-1) (Fig. 1), has been shown to play an important role in diseases such as arteriosclerosis, type 2 diabetes mellitus, neurodegenerative diseases, gout, arthritis and silicosis [1]. Priming of the NLRP3 inflammasome is a prerequisite for its activation. Priming can upregulate NLRP3 、pro-caspase-1、pro-IL-1β and pro-IL-18 expression, and induce post-translational modifications of NLRP3(such as ubiquitination, phosphorylation, and SUMOylation) [7–9]. The priming is achieved through the identification of pathogen-associated molecular patterns and damage-associated molecular patterns. Bauernfeind et al. confirmed that TNF, IL-1, HMGB1, IFN, TGF-1β and LPS could upregulate NLRP3 expression by inducing nuclear factor-κB (NF-κB) activation [10, 11]. Primed NLRP3 induces inflammasome assembly and full activation upon recognition of appropriate activator. The activators include K^+^ efflux, Cl^−^ efflux, Ca2^+^ mobilization, lysosome destruction, anti-Golgi dissociation, mitochondrial dysfunction, mitochondrial ROS production and mitochondrial DNA release into the cytosol [12, 13]. There is mounting evidence that mitochondria are not only involved in the regulation of NLRP3 inflammasome activation, but also the docking site of inflammasome assembly. Once activated, NLRP3 associates with both mitochondria and mitochondria associated membranes. Cardiolipin, mitochondrial antiviral signaling protein and mitomycin 2 are considered to be the connection points between NLRP3 and mitochondria, playing important roles in NLRP3 localization and activation [14, 15]. These processes are called Canonical activation of the NLRP3 inflammasome pathways (Fig. 2).

**Fig. 3:**
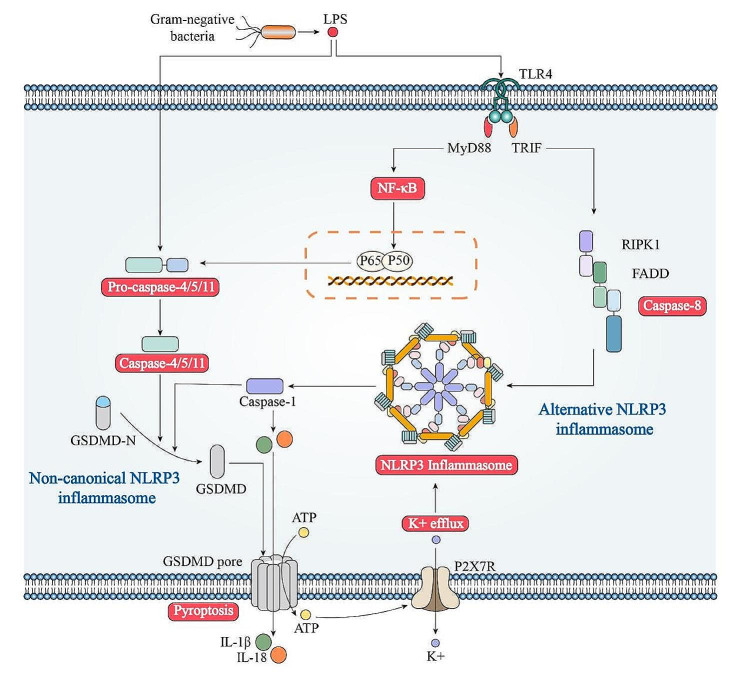
Noncanonical and alternative activation of the NLRP3 inflammasome pathways.Noncanonical activation of NLRP3 inflammasome is induced by gram-negative bacteria. Release of LPS from engulfed bacteria into the cytoplasm activates human Casp4/5 or mouse Casp11, which cleaves GSDMD to induce pyroptosis and indirectly activates the NLRP3 inflammasome to activate Casp1 and IL-1b and IL-18. Noncanonical activation does not require priming as Casp4 is present at a high level. The alternative pathway of activation is elicited by TLR4 agonists like LPS, which activates the TLR4-TRIF-RIPK1-FADD-Casp8 signaling. Casp8 activates the NLRP3 inflammasome but does not require K1 efflux, ASC speck formation, or pyroptosis

However, findings in recent years have revealed that NLRP3 inflammasome activation does not always follow a two-step activation pattern. The LPS released by Gram-negative bacteria can activate human caspase-4/5 or mouse caspase-11 to lyse GSDMD and induce pyroptosis.Activated caspase-11 activates pannexin-1 to release ATP and induce K + efflux, thus mediating the activation and oligomerization of NLRP3.And caspase-4 is highly expressed in human cells, so its activation does not need priming [16, 17]. This process is called Noncanonical activation of the NLRP3 inflammasome pathways (Fig. 1).Hornung et al. found another NLRP3 inflammasome activation pathway in human monocytes: mediating TLR4-TRIF-RIPK1-FADD-CASP8 signaling could activeNLRP3 inflammasome and release mature IL-1β, but did not lead to pyroptosis [18]. Lin et al. observed that simultaneous stimulation of TLR and NLRP3 by pathogenic microorganisms could lead to rapid assembly of NLRP3 inflammasome and pyroptosis in mice macrophages, which depends on the TLR signaling molecule IL-1 receptor-associated kinase and its kinase activity [19]. This process is called alternative activation of the NLRP3 inflammasome pathways (Fig. 3).

In addition, NLRP3 works with other sensors to co-activate the inflammasome. Freeman et al. found that lysophosphatidylcholine could induce NLRC4 and NLRP3 to form different inflammasomes in microglia and astrocytes, synergistically promote the mature IL-1β release, and play an important role in its hyperplasia [20]. Kalantari et al. demonstrated that Plasmodium and Aspergillus could activate NLRP3 and AIM2 inflammasomes, forming a dual cytoplasmic surveillance system [21, 22]. Davis et al. confirmed that NLRC5 bound to NLRP3 in an NBD-dependent but LRR-inhibitory manner in the presence of pathogens, and the two co-activated the inflammasome [23]. NIMA-associated kinase 7 is demonstrated not to interact with other inflammasome sensors (such as NLRC4 and AIM2), but to specifically interact with NLRP3 by polymerizing into complexes essential for ASC spot formation and caspase-1 activation, suggesting that NEK7 is a core component unique to the NLRP3 inflammasome [24–26]. In addition, Dos observed strong colocalization of NLRP3 and vimentin in activated alveolar macrophages, and the colocalization between NLRP3 and ASC was absent after vimentin knockdown, suggesting that vimentin is associated with components of the inflammasome [27]. Lang et al. reported that the activated macrophage migration inhibitory factor could not only regulate the interaction of NLRP3- vimentin, but also interact with NLRP3 and directly participated in the NLRP3 inflammasome assembly and activation, indicating that migration inhibitory factor’s role in inflammasome activation is independent of its role as a cytokine [28]. Recently, the proteins which interact with NLRP3 inflammasome complex but are not its components have been successively reported. Such as Pyrin-only proteins (POPs, also known as PYDC proteins) and Card-only proteins (COPs). POPs include POP1-POP4. Of these, POP1 and POP2 combine with ASC, thus inhibiting the interaction between NLRP3 and ASC and preventing the over-activation of NLRP3 [29, 30]. In human COPS, COP1, ICOBERG and INCA can bind to full-length caspase-1, thus preventing its own activation and limiting inflammasome activation [29] (Fig. 2).

##  NLRP3 inflammasome induces pyroptosis and its inhibitors

**Fig. 4:**
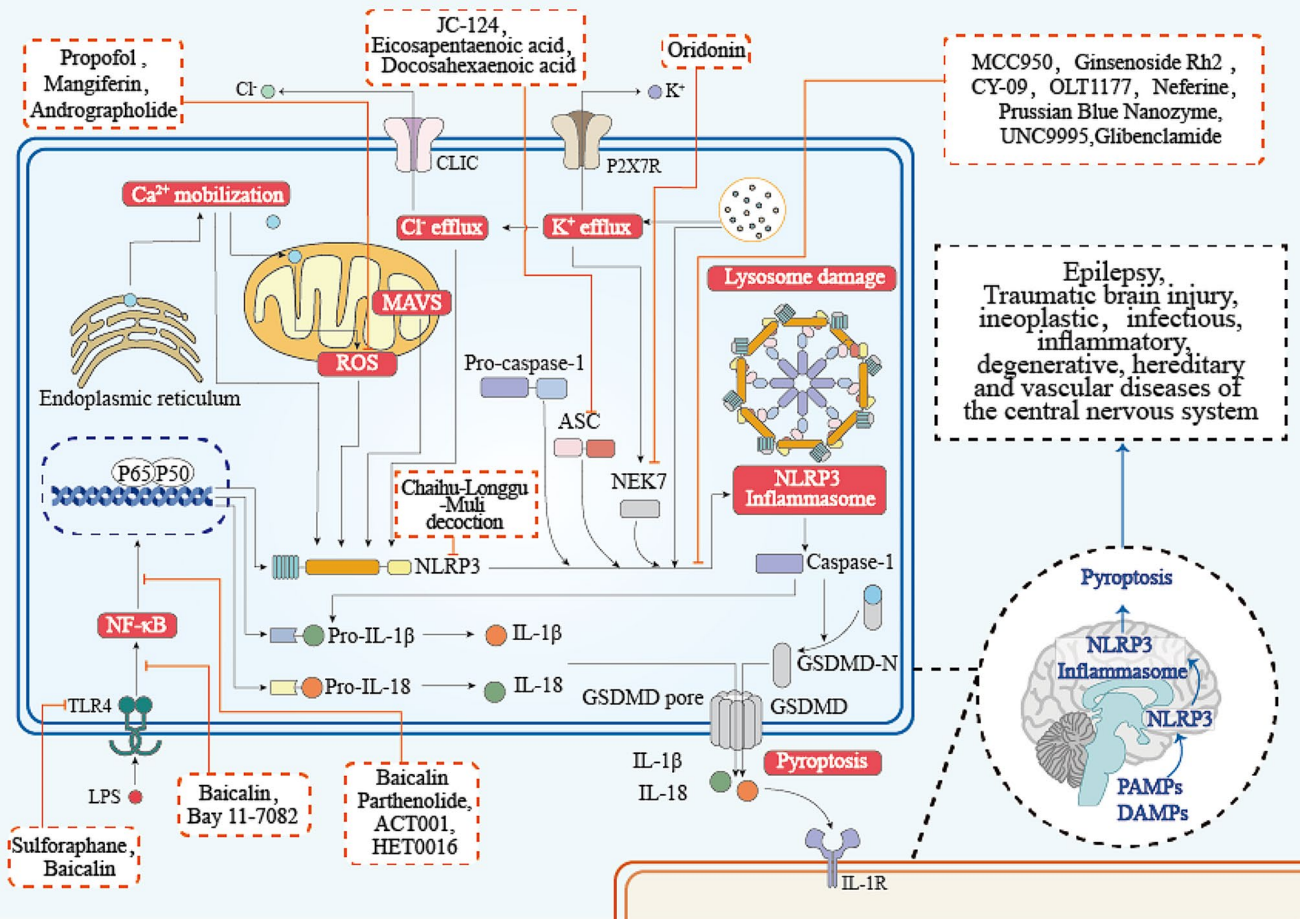
NLRP3 inflammasome signal path inhibitors in experimental models of central nervous system diseases. MCC950 inhibited NLRP3 activation and ATPase activity. JC-124 prevented the oligomerization in the interaction between NLRP3 and ASC. Baicalin inhibited the upregulated expression of TLR4, suppressed the phosphorylation and degradation of IκBα, and inhibited the subsequent nuclear translocation of NF-κB p65. Parthenolide curbed the phosphorylation of NF-κB. Ginsenoside Rh2 inhibited the NLRP3 activation. CY-09 prevented NLRP3 oligomerization. OLT1177 inhibited ATPase activity. Oridonin blocked the interaction between NLRP3 and NEK7, thereby inhibiting NLRP3 inflammasome assembly and activation. Bay 11-7082 curbed the phosphorylation of NF-κB. Neferine inhibited NLRP3 activation. Docosahexaenoic acid reduced the expression of NLRP3.Propofol depressed the TXNIP expression, oxidative stress, and NLRP3 inflammasome activation. Mangiferin reduced the expression of TXNIP, depress the oxidative stress and enhance the endogenous antioxidants. ACT001 restrained NF-κB nuclear translocation in microglia cells through inhibiting AKT phosphorylation. HET0016 inhibited the phosphorylation of p38 MAPK. Prussian Blue Nanozyme inhibited the assembly and activation of NLRP3 inflammasome.UNC9995 inhanced β-arrestin2 interacting with NLRP3 to interfere inflammasome assembly. Glibenclamide inhibition of NLRP3 activation. Andrographolide promoted the parkin-dependent autophagic flux formation in microglia; resulting in the removal of defective mitochondria which in turn inhibit NLRP3 inflammasome activation

Activation of NLRP3 inflammasome can induce caspase-1-dependent pyroptosis in host cells. Gasdermin D is a mediator of pyroptosis. After interacting with caspase-1, it can bind to phosphatidylinositol phosphates and phosphatidyl serine in the membrane leaflets, aggregate and insert into the plasma membrane to form a pore. Inflammatory cytokines such as IL-1β and IL-18 are released into the interstitial space and activate corresponding receptors in adjacent cells, causing an immune cascade and amplifying the injury effect [31] (Fig. 2).There are more and more reports on NLRP3 as a therapeutic target for a variety of diseases in recent years.MCC950, a sulfonylurea compound, is the most effective and specific inhibitor of NLRP3, and has shown promising therapeutic effects in various preclinical immunopathological models [1], including CAPS, experimental autoimmune encephalomyelitis, Alzheimer’s disease, traumatic brain injury, arrhythmias, myocardial infarction, diabetic encephalopathy, HANDs, acute pancreatitis, spinal cord injury, brain edema, cerebral ischemia-reperfusion injury, brain injury following intracranial haemorrhage, age-related osteoporosis, neonatal hypoxic-ischemic encephalopathy, post-stroke cognitive impairment and other diseases [5, 32]. Furthermore, the inhibitor of cystic fibrosis transmembrane conductance regulator C172 and its analogues CY-09, oridonin have also been shown to specifically inhibit NLRP3 inflammasome [2, 4, 33] (Table 1; Fig. 4).

**Table 1:** NLRP3 inflammasome signal path inhibitors in experimental models of central nervous system diseases

	Effects	Mechanism	Animal model(Dose, ad- ministration)	Refs.
MCC950	①Alleviated CUMS‑induced neuron injury and AD‑like pathological changes ②Improve cognitive function by attenuating autophagy in neuronal cells.	Inhibition of NLRP3 activation and ATPase activity	Male C57BL/6 mice(10 mg/kg, i.p.)	[101, 102, 156]
JC−124	Decreased multiple AD pathologies	Prevention of oligomerization in the interaction between NLRP3 and ASC	Female APP/PS1 mice(50 and 100 mg/kg, gavage)	[100]
Baicalin (BAI)	①Uppressed microglial activation and proinflammatory cytokine levels, prevented neuronal apoptosis, ameliorated learning and memory deficits in APP/PS1 mice ②Reversed MPTP-induced motor dysfunction, loss of dopaminergic neurons, and proinflammatory cytokine elevation	Inhibited the upregulated expression of TLR4, suppressed the phosphorylation and degradation of IκBα, and inhibited the subsequent nuclear translocation of NF-κB p65	①APP/PS1 transgenic mice(103 mg/kg) ②Male C57BL/6J mice (140, 280, and 560 mg/ kg, i.g.)	[103, 115]
Parthenolide (PTN)	Relieved neural function deficits, brain edema and neuron apoptosis and improved the memory and learning function of TBI mice.	Curbed the phosphorylation of NF-κB	C57BL/6 mice (1 mg/kg, i.p.)	[48]
Ginsenoside Rh2 (GRh2)	Alleviated neuropathological damage and neuronal apoptosis in cortical tissue of T. gondii-infected mice.	Inhibition of NLRP3 activation	Female BALB/c mice (100 mg/kg, gavage)	[44]
CY−09	①Ameliorated seizure severity and inhibited PTZ-induced neuronal loss by attenuating the activation of astrocytes and the secretion of IL−1β ②Improve AD pathology and relieve cognitive impairment in these mice	Prevention of NLRP3 oligomerization	①Male C57BL/6 mice(2.5 mg/kg, i.p.) ②female C57BL/6J mice( 2.5 mg/kg, i.p.)	[104, 157]
OLT1177	①Protected against functional deficits and demyelination in the spinal cord ②Prevented the loss of motor function, reduced the levels of α‑synuclein, modulated pro‑inflammatory markers in the nigrostriatal areas of the brain, and protected dopaminergic neurons from degeneration in the MPTP model of PD	Inhibition of ATPase activity	①Female adult C57BL/6(60 mg/kg, gavage) ②Male wild- type C57BL/6J mice(i.p.)	[63, 167]
Oridonin	Improved functional im- pairments and neuro- pathological changes in animals with TBI	Blocked the interaction between NLRP3 and NEK7, thereby inhibiting NLRP3 inflammasome assembly and activation	Female mice, SPF grade(20 mg/kg, i.p.)	[168]
Bay 11-7082	Relieved neural function deficits, brain edema and neuron apoptosis and improved the memory and learning function of TBI mice.	Curbed the phosphory- lation of NF-κB	C57BL/6 mice (1 mg/kg, i.p.)	[48]
Neferine	Reduced seizure severity, attenuated neuronal loss, inhibited excess glutamate, and suppressed inflammation in the hippocampi of seizure rats.	Inhibition of NLRP3 activation	Male Sprague- Dawley rats(10 and 50 mg/kg, i.p.)	[154]
Eicosapentaenoic acid	Alleviate PTZ-induced seizure and depressive-like behavior	Inhibited the binding of NLRP3 and ASC	Male ICR mice(diet contain- ing EPA ethy- lester (EPA = 1%, w/w)	[169]
Docosahexaenoic acid	Alleviate PTZ-induced seizure and depressive-like behavior	Decreases the protein levels of ASC and Caspase−1	Male ICR mice(diet contain- ing DHA ethy- lester (DHA EPA = 1%, w/w)	[169]
Chaihu-Longgu-Muli decoction	Suppressed the frequency and duration time of epileptic seizures by improving pyroptosis in hippocampal neurons of TLE rats.	Reduce the expression of NLRP3	Male Sprague-Dawley rats(25 g/kg, gavage)	[158]
Propofol	Relieved the inflammatory response and attenuate brain in jury	Depress the TXNIP expression, oxidative stress, and NLRP3 inflammasome activation	Male SPF Sprague–Dawley rats (50 mg/kg, i.p.)	[49]
Mangiferin	Amelioration of cerebral cortex damage was found in rats suffering bTBI	Reduced the expression of TXNIP, depress the oxidative stress and enhance the endogenous antioxidants	Male adult Sprague-Dawley rats(100 mg/kg, i.p.)	[50]
ACT001	Relieved the extent of blood-brain barrier integrity damage and alleviated motor function deficits after TBI via reducing traumainduced activation of microglia cells	Restrained NF-κB nuclear translocation in microglia cells through inhibiting AKT phosphorylation	Male C57BL/6 mice(100 mg/kg, gavage)	[51]
HET0016	Reduced the lesion volume; neuronal death	Inhibited the phosphory- lation of p38 MAPK	Male Sprague–Dawley rats (1 mg/kg, i.p.)	[52]
Prussian Blue Nanozyme	Alleviated motor deficits and rescued dopaminergic neuron loss induced by MPTP	Inhibited the assembly and activation of NLRP3 inflammasome	C57BL/6J mice(3 µL, 1 mg/ ml, ICV administration)	[111]
UNC9995	Rescued the TH + neurons loss and inhibited glial cells activation in mouse substantia nigra in a Drd2 dependent manner	Enhanced β-arrestin2 interacting with NLRP3 to interfere inflammasome assembly	C57BL/6J mice (2 mg/kg, i.p.)	[113]
Glibenclamide	Ameliorated *P* + M-induced degeneration of dopaminergic neurons and motor function	Inhibition of NLRP3 activation	Male C57BL/6 mice (1 mg/kg, i.p.)	[114]
Andrographolide	Rescued the dopaminergic neuron loss and improved the behavioural parameters in animal model	Promoted the parkin-dependent autophagic flux formation in microglia; resulting in the removal of defective mitochondria which in turn inhibit NLRP3 inflammasome activation.	C57BL/6 male mice(5 mg and 10 mg /kg, i.p.)	[116]

##  The role of NLRP3 inflammasome in CNS diseases

###  CNS infection

####  Viral infections of the CNS

NLRP3 inflammasome can recognize various pathogen-related molecular patterns and danger-related molecular patterns during viral replication, and is a key player in the organism’s antiviral response. Currently, NLRP3 has been found to be localized mostly in microglia, and also expressed in other brain cells such as oligodendrocytes, brain endothelial cells and astrocytes [34–36]. Zika virus (ZIKV) is a neurotropic virus, which can not only break through the BBB and invade the CNS, but also damage peripheral nerves. It can also be transmitted vertically from mother to her fetus, and pose a serious threat to fetal health. Gim et al. found that ZIKV NS5 protein combined with Nacht and LRR domains of NLRP3 in cytoplasm to form a NS5-NLRP3-ASC globular structure, which promoted NLRP3 inflammasome activation and IL-1β release, and induced aggressive inflammation response [37]. Knockdown of NLRP3 can inhibit ZIKV-mediated IL-1β secretion in mice, indicating that negative regulation of NLRP3 inflammasome or preventing NS5 binding to NLRP3 and ASC can reduce the damage caused by ZIKV infection-induced aggressive inflammatory reaction. Since 2007, large-scale outbreaks of hand, foot and mouth disease caused by enterovirus have occurred continuously in China. The dominant prevalence of enterovirus 71(EV71) has led to severe CNS complications and death. A study has demonstrated that EV71 3D RNA polymerase in macrophages and peripheral blood monocytes can directly interact with NLRP3 to form 3D-NLRP3-asc circular structure, promote NLRP3 inflammasome assembly and activate IL-1β [38]. XIAO et al. confirmed that VIM mediated NLRP3 inflammasome activation in EV71-infected mice and promoted brain inflammation and neuronal injury, but failed to verify whether 3D-NLRP3-asc ring structure was formed in the CNS [39]. Japanese encephalitis virus is a common cause of acute epidemic viral encephalitis. ROS production and K^+^ efflux mediated by Japanese encephalitis virus infection can induce NLRP3 inflammasome assembly, Caspase-1 activation and IL-1β release [40]. Taken together, negative regulation of NLRP3 inflammasome can reduce the damage caused by CNS virus infection-induced aggressive inflammatory response.

####  Bacterial infections of the CNS

Streptococcus pneumoniae (*S. pneumoniae*) activates NLRP3 through its virulence factor pneumolysin [41]. Knockdown of NLRP3 and ASC attenuates brain inflammation in the mice model of *S. pneumoniae* meningitis [42]. Similarly, Kim et al. found that *S. pneumoniae* could induce caspase-1 activation and downstream inflammatory cytokines maturation in a mice meningitis model, suggesting that NLRP3 inflammasome plays a key role in this process, and inhibition of inflammasome activation is a practical strategy for the treatment of *S. pneumoniae* meningitis [43].

####  Other pathogen infections of the CNS

Kalantari et al. [21, 22] demonstrated that Plasmodium and Aspergillus could activate NLRP3 and AIM2 inflammasomes to form a dual-cytoplasmic surveillance system.Lee et al. found that Mycobacterium tuberculosis can stimulate microglia-leukocyte interaction, and act as a priming signal to activate NLRP3 inflammasome of microglia and induce IL-1β maturation in patients with tuberculous meningitis. Jin et al.found that Ginsenoside Rh2 can inhibit the TLR4/NF-κ b signaling pathway, block the activation of NLRP3 inflammasome, and improve the neuronal damage induced by Toxoplasma gondii infection [44].

###  Traumatic brain injury

Traumatic brain injury (TBI) is a change in brain function caused by external mechanical forces on the head or neck, and it is one of the most common causes of death and disability [45]. Peripheral edema is the main mechanism of secondary brain injury after craniocerebral injury. Yi et al. found that NLRP3 inflammasome expression increased in mice after TBI, and NLRP3 gene knockdown could alleviate brain edema, suggesting that NLRP3 inflammasome plays an important role in regulating brain edema and secondary inflammation after TBI [46]. Xu et al. detected that NLRP3 inflammasome expression was up-regulated in the cortex surrounding brain injury, especially in microglia. MCC950 could alleviate brain edema, reduce lesion volume, and decreases cell death in TBI mice, thereby reducing the severity of brain injury and showing a good neuroprotective effect [47]. However, this effect disappeared in mice with depleted microglia, indicating that NLRP3 inflammasome is involved in the inflammatory response to TBI, and MCC950’ s specific inhibition of NLRP3 inflammasome may be a promising therapeutic approach for TBI.In addition, JC124, HET0016, ACT001, pathenolide, mangiferin, propofol and other drugs have been reported to reduce the activation of NLRP3 inflammasome and caspase-1 around injury in TBI mice, and alleviate neurological deficits and neuronal damage [48–52].

###  Immunoinflammatory diseases of the CNS

####  Anti-NMDAR encephalitis

Autoimmune encephalitis has broadly come to mean a kind of nerve dysfunction is triggered by auto-specific antibodies attacking the CNS and mediating inflammatory brain parenchyma lesions. Its prevalence is increasing year by year. Anti-N-methyl-d-aspartate receptor (NMDAR) encephalitis is the main type of autoimmune encephalitis, which progresses rapidly. Some patients still have serious neurological deficits or mental disorders even after standardized treatment, seriously affecting the health and quality of life of patients [53]. Peng et al. observed that NLRP3 and IL-1β in cerebrospinal fluid were increased significantly in patients with anti-NMDAR encephalitis, and they were positively correlated with each other. Further follow-up showed that the decrease of maximum modified Rankin scale was positively correlated with the decrease of NLRP3 inflammasome in cerebrospinal fluid in patients with anti-NMDAR encephalitis [54]. It is suggested that NLRP3 inflammasome and its downstream inflammatory cytokines are involved in the neuroinflammatory process of anti-NMDAR encephalitis. its level reflects the severity of anti-NMDAR encephalitis patients, and can help judge the severity and prognosis of anti-NMDAR encephalitis.

####  NLRP3 autoimmune GFAP astrocytopathy

Autoimmune glial fibers acidic protein (GFAP) astrocytopathy is an autoimmune meningitis/encephalomyelitis mediated by GFAP antibody. At present, there are no recognized diagnostic criteria and treatment guidelines for it, and it is difficult to diagnose and treat. Luo et al. observed that the levels of NLRP3 inflammasome and its downstream cytokines IL-1β, IL-6 and IL-17 were significantly increased in cerebrospinal fluid of patients with GFAP astrocytic disease, and NLRP3 expression was positively correlated with cytokines and disease severity. Moreover, they found that NLRP3 and inflammatory cytokines levels were significantly positively correlated with anti-GFAP antibody titers and disease severity, indicating that NLRP3 inflammasome is activated significantly in patients with autoimmune GFAP astrocytopathy, triggers downstream inflammatory response, and participates in the occurrence of the disease [55]. These results suggest that NLRP3 inflammasome may be a new target for evaluating the severity and treatment of autoimmune GFAP astrocytopathy.

####  NLRP3 neuromyelitis optica spectrum disorders

Neuromyelitis optica spectrum disorders (NMOSDs) are a group of CNS inflammatory demyelinating diseases mediated by antigen-antibody. Patients who do not receive immunosuppressive therapy have poor prognosis and high mortality [56]. Peng et al. found that the levels of NLRP3, mtDNA, IL-1β, IL-6, and IL-17 in cerebrospinal fluid of NMOSDs patients were higher than those of the control group, and the EDSS scores of NMOSDs patients at the relapse stage was positively correlated with NLRP3 and mtDNA in CSF [57], suggesting that NLRP3 inflammasome-mediated pyroptosis after mitochondrial injury may play an important role in the pathogenesis of NMOSDs.

####  Multiple sclerosis

Multiple sclerosis (MS) is an autoimmune inflammatory demyelinating disease of CNS known as the most common CNS disease which leads to the disability of young and middle-aged people [58]. The expression of NLRP3 and IL-1β was increased in microglia of MS patients, and the percentage of NLRP3 and IL-1β positive CNS cells was positively correlated with demyelinating activity and imaging severity [59]. The activated NLRP3 inflammasome promotes the release of a large amount of mature IL-1β into the extracellular space, causing a cascade of inflammatory reactions and aggravating the progression of MS [60]. Using specific NLRP3 inflammasome inhibitors can alleviate axonal injury and disease severity in experimental autoimmune encephalomyelitis mice [59]. Using interferon-β can inhibit IL-1β release and reduce NLRP3 inflammasome activity, thus shortening the course of MS [61]. Additionally, Voet et al. found that deficiency of NF-κB regulatory protein A20 in microglia aggravated MS-like disease. The reason for this was that A20 deficiency caused NLRP3 inflammasome hyperactivation, leading to increased secretion of IL-1β [62]. OLT1177, a selective NLRP3 inflammasome inhibitor, ameliorates neurological decline and nerve tissue damage in EAE mice, demonstrating its beneficial role in the treatment of MS [63]. The aforementioned studies indicate that NLRP3 inflammasome and IL-1β are potential prognostic biomarkers and potential therapeutic targets for MS. Inhibiting the activities of NLRP3 and downstream inflammatory cytokines such as IL-1β may help attenuate persistent inflammatory response in CNS and s slow disease progression.

###  Stroke

Stroke is a kind of disorder of cerebrovascular circulation caused by various factors, which leads to ischemic and hypoxic necrosis of brain tissue. It is usually divided into hemorrhagic stroke and ischemic stroke [64]. With the rapid development of global population aging and the rising prevalence of risk factors such as obesity, hypertension, diabetes and hyperlipidemia, the stroke incidence is increasing year by year and the age of onset is younger, which seriously threatens human health [65]. Under the existing medical technology and conditions, its morbidity, disability and mortality are extremely high。 And it is the second largest cause of death and the third largest cause of disability among adults in the world [66]. Therefore, it is a major clinical challenge to find new treatment targets and formulate new treatment strategies to reduce its disability and mortality .

####  Ischemic stroke and cerebral ischemia-reperfusion injury

Ischemic stroke accounts for more than 80% of all stroke patients, which poses a serious threat to human health [67]. The key to treatment is to restore cerebral blood flow perfusion in ischemic area as soon as possible. However, cerebral blood flow reperfusion may aggravate ischemic tissue injury and inflammation, resulting in some ischemic brain tissue injury or dysfunction (i.e., cerebral ischemia-reperfusion injury) [68]. It has been confirmed that knockdown of NLRP3 gene can reduce cerebral infarct volume, attenuate brain edema, maintain BBB permeability, and decrease nerve cell death in mice with ischemic stroke [69]. Using MCC950 can improve the neurological deficit and enhance the long-term survival rate but not reduce the cerebral infarct volume of diabetic mice with transient middle cerebral artery occlusion, which may be due to the inhibitory effect of MCC950 is not as complete as NLRP3 knockdown [68]. Currently, the important roles of NLRP3 inflammasome assembly, caspase-1 activation, IL-β and other downstream inflammatory cytokines release have been confirmed in cerebral ischemia/reperfusion injury after ischemic stroke. Meanwhile, the effect of NLRP3 inflammasome related signaling pathways on this disease has also drawn more and more attention. Hou et al. found that up-regulating Nrf2 could lead to decreased expression of TXNIP, NLRP3 inflammasome, Caspase-1, IL-18, and IL-1β, and the protective effect of Nrf2 was basically eliminated after knockdown of Trx1 in the mice model of middle cerebral artery occlusion, suggesting that Nrf2 can inhibit NLRP3 inflammasome activation by regulating Trx1/TXNIP complex [70]. In addition, An et al. reported that histidine play a neuroprotective role by inhibiting NLRP3-mediated pyroptosis by modulating AMPK/GSK3β signaling pathway [71]. He et al. found that ATF4 gene overexpression can up-regulate Parkin expression, enhance phagocytic activity, and inhibit inflammatory response mediated by NLRP3 inflammasome. Knockdown of Parkin gene effectively reversed the above process, confirming that ATF4 can reduce cerebral ischemia-reperfusion injury by inhibiting NLRP3 inflammasome activation, and the mechanism may correlate with the inhibition of Parkin-dependent mitotic activity [72]. There are some studies have confirmed that meisoindigo and anthocyanin from myrica rubra also can inhibit NLRP3 inflammasome activation and regulate microglia/macrophage polarization by regulating TLR4/NF-κB signaling pathway, thus playing a neuroprotective role in ischemic stroke and cerebral ischemia-reperfusion injury [73, 74].

####  Hemorrhagic stroke

Although the incidence of hemorrhagic stroke is lower than that of ischemic stroke, its symptoms are more severe, the rate of disability and mortality are higher. Intracerebral hemorrhage(ICH) is the most common subtype of hemorrhagic stroke, with a 28-day mortality rate as high as 47%, which is much higher than that of ischemic stroke (3%) [67]. The catastrophic outcome of intracerebral hemorrhage is the result of local brain parenchyma destruction caused by hematoma and the secondary brain injury caused by BBB dysfunction, brain edema around hematoma, nerve inflammation and so on [75]. It was found that NLRP3, caspase-1, and IL-1β levels in CD1mice microgliaa increased significantly at 3 h after ICH. Knocking down NLRP3 by siRNA could reduce IL-1 β level and neutrophil infiltration around the hematoma [76], indicating that NLRP3 is an important participant in neuroinflammation after ICH. Overactivated NLRP3 in choroid plexus epithelium after ICH can regulate cerebrospinal fluid secretion through ion cotransporter NKCC1 and participate in the occurrence and development of hydrocephalus after ICH. Knocking out NLRP3 or applying MCC950 can reduce the secretion of cerebrospinal fluid and alleviate the neurological deficit and hydrocephalus [77]. Activated M2 microglia can help hematoma clearance by phagocytosis of red blood cells and tissue fragments. MitoQ can inhibit NLRP3 activation by reducing the production of mitochondrial ROS, and promote the transformation of microglia to M2 phenotype, thus reducing brain edema and neuroinflammation after ICH, promoting hematoma clearance, and improving the prognosis of ICH experimental animals [78]. By inhibiting the activation of NLRP3 and partially maintaining the integrity of the BBB after ICH, OLT1177 can reduce vascular leakage, reduce neuronal loss and brain edema in the damaged hemisphere, and effectively alleviate neurological impairment [79]. Didymin can attenuate microglial apoptosis, neutrophil infiltration, brain edema and BBB damage after ICH by up-regulating Rkip to inhibit the assembly of NLRP3 inflammasome, and improve neurological impairment [80]. Ursolic acid can down-regulate NLRP3 expression by inhibiting NF- κ B phosphorylation, thus reducing BBB destruction, perihematoma edema and neuronal loss after ICH [81]. At the same time, some studies have noted that the number of Helicobacter pylori in the gut of mice increased after ICH. After intraperitoneal injection of MCC950, the proportion of bacteroides, bifidobacteria and Bacteroides in the gut of mice increased, while the Helicobacter pylori decreased, and the brain edema and neurological impairment around hematoma were improved [82]. Unfortunately, this study did not deeply explore the bidirectional regulation and causal relationship between intestinal microflora, NLRP3 inflammasome and cerebral hemorrhage after ICH. In addition, silymarin, atorvastatin, baicalein, mst4, h2s, a68930-induced activation of drd1, fimasartan, p2y6 receptor activation, histone deacetylation 10, verbascoside, verapamil, cordycepin, dexmedetomidine, andrographolide and others can directly or indirectly inhibit NLRP3 activation to reduce nerve inflammation, improve secondary brain injury after ICH, and play a neuroprotective role [83–96].

- Subarachnoid hemorrhage (SAH) is the second most common subtype of hemorrhagic stroke. Early brain injury characterized by brain edema, delayed cerebral ischemia caused by cerebral vasospasm, and persistent neuroinflammation after SAH are considered to be associated with adverse outcomes [83]. It has been found that the expressions of NLRP3, ASC and active caspase-1 in monocytes of aneurysmal subarachnoid hemorrhage patients are significantly increased, and MCC950 can effectively block the release of IL-1 β, IL-18 and tissue factor mediated by excessive activation of NLRP3 inflammasome [84]. A number of experiments on animal models of SAH further confirmed that red blood cells dissolved in the subarachnoid space after SAH event, oxidative stress and endoplasmic reticulum stress triggered by hemolytic products, potassium efflux and extracellular accumulation of ATP mediated the assembly of NLRP3 inflammasome. Inhibition of NLRP3 activation can effectively improve early brain injury and delayed cerebral ischemia after SAH [85–87]. The relationship between the overactivation of NLRP3 and the adverse outcome of SAH has been fully verified. NLRP3 is undoubtedly a potential target for SAH therapy. Studies have confirmed that MCC950 can effectively improve brain edema, BBB destruction, neutrophil infiltration, microthrombosis, neurological dysfunction and delayed cerebral vasospasm after SAH by inhibiting NLRP3 activation to reduce the polarization of microglia [87]. Low-dose fimasartan can significantly reduce the activation of NLRP3/ASC/caspase-1/NF- κ B pathway after ICH, thus alleviating the cerebral edema and improving neurological function [88]. INT-777, a TGR5 agonist, can inhibit the NLRP3 activation through TGR5/cAMP/PKA signaling pathway, alleviate acute cerebral edema and neuroinflammation, and hippocampal neuronal degeneration 28 days after SAH [89]. Inhibition of NLRP3 activation by PHLDA1 can induce M2 polarization of microglia after SAH and improve neuroinflammation and neuronal apoptosis [90]. Takinib can reduce oxidative damage, neuroinflammation, brain edema and neuronal apoptosis after SAH by inhibiting TAK1-ROS-NLRP3 signaling pathway, and improve neurological deficit [91]. In addition, resveratrol, schisandrin B, fluoxetine, melatonin and pterostilbene have also been reported to play a protective role in SAH by inhibiting the activation of NLRP3 [92–95]. To sum up, targeting NLRP3 is a potential therapeutic strategy to improve the adverse outcome of SAH.

### Degenerative diseases of the CNS

####  Alzheimer’s disease

Alzheimer’s disease (AD) is a neurodegenerative disease manifested by neurological degeneration and cognitive decline, resulting from the amyloid-β (Aβ) protein accumulation, Neurofibrillary tangles formed by hyperphosphorylated tau, and long-term persistent aseptic neuroinflammation, which is the first cause of dementia [96]. There is still no widely recognized drug for preventing or treating AD on the market, despite a great deal of effort has been put in it. It was found that amyloid-β protein, Aβ oligomers, and hyperphosphorylated tau can activate NLRP3 inflammasome [97, 98]. The activated NLRP3 inflammasome in microglia can promote IL-1β maturation, induce pyroptosis, and increase amyloid-β protein accumulation and tau protein phosphorylation [97, 99]. In conclusion, NLRP3 inflammasome plays an important role in the occurrence and development of AD. Blocking NLRP3 inflammasome assembly or specifically inhibiting its downstream inflammatory cytokine IL-1β is an effective strategy to reduce the deterioration of AD neuronal function. At present, NLRP3 inhibitors MCC590, JC124, OLT1177 ,CY-09, andbaicalin have shown therapeutic effects in AD animal models, effectively improving neurological degeneration and cognitive decline in AD mice, but their safety and therapeutic effect in AD patients have not been validated [100–104].

####  Parkinson’s disease

Parkinson’s disease (PD) is a neurodegenerative disease manifested by movement disorders, tremor and balance disorders. Its main pathological features are progressive loss of dopaminergic neurons in the substantia nigra pars compacta, Lewy bodies and Alpha-synuclein (α-Syn) accumulation, and excessive neuroinflammation [105]. Several studies have shown that accumulated α-Syn can activate the NLRP3 inflammasome. Zhou et al. found that the intracellular ROS production and lysosomal cathepsin B release increased after microglia ingested α-Syn, thereby activating NLRP3 inflammasome and promoting IL-1β and IL-18 maturation [106]. Panicker et al. found that α-Syn could not only activate NLRP3 but also induce NF-κB-p65 nuclear translocations to promote NLRP3 priming. In addition, they confirmed that α-Syn uptake by microglia was co-regulated by Fyn kinase and CD36, and α-Syn mediated NLRP3 priming and activation was also regulated by Fyn kinase. NLRP3 inflammasome assembly activates caspase-1, and the activated caspase-1 directly cleaves a-Syn to increase its aggregation tendency. syn-induced inflammasome priming and activation and inflammasome-mediated a-Syn truncation and aggregation form a vicious circle, thus eventually leading to neuronal death [107]. Therefore, inhibition of NLRP3 inflammasome assembly, caspase-1 activation and the release of downstream inflammatory cytokines are important means to protect neural function. In recent years, several studies have found that baicalein, andrographolide, berberine, Genkwanin, Dl-3-n-Butylphthalide, glibenclamide, UNC9995(Drd2 biased agonist), Dopamine, Prussian Blue Nanozyme, KPT-8602 (XPO1 Inhibitor), Cordycepin, long noncoding RNA (HOTTIP), Long noncoding RNA GAS5, Long-Noncoding RNA LncZFAS1 and others can improve the neurobehavioral function of Parkinson’s disease mice by inhibiting NLPR3 inflammasome activation, showing good efficacy [108–119]. However, there is still a long way from animal experiments to clinical applications.

####  Amyotrophic lateral sclerosis

Amyotrophic lateral sclerosis (ALS) is a kind of progressive motor neuron degenerative disease characterized by Skeletal muscle weakness and atrophy from degeneration of motor neurons in cortex, brainstem and spinal cord [120]. Its etiology is still unknown. About 10% of ALS cases can be attributed to genetic mutations, among which copper-zinc superoxide dismutase (SOD1) gene mutation is the first discovered and the most studied mutation [121]. Misfolding of specific proteins is at the heart of ALS. And the SOD1 mutant protein, a major component of some familial and sporadic ALS protein deposits, spreads its misfolded conformation like a prion [122, 123]. Johann et al. found that the expression of NLRP3 and ASC increased in astrocytes of SOD1^G93A^ALS model mice and postmortem spinal cord tissues of ALS patients [35]. Deora et al. further discovered that endocytosis of aggregated or soluble SOD1G93A mutant protein in microglia of SOD1^G93A^ALS model mice could increase ROS production, promote Caspase-1 and IL-1β cleavage, ASC spot formation and IL-1β secretion. Of these the aggregation SOD1 protein had a more significant promoting effect. The specific NLRP3 inhibitor MCC950 can block IL-1β secretion, proving that NLRP3 inflammasome plays a key role in SOD1 mutant protein-induced IL-1β secretion in microglia [124]. The existing treatments have not been effective in changing the trajectory of ALS and have only modestly improved survival. Therefore, inhibition of NLRP3 activation may be a potential way to slow down ALS proliferative neuroinflammatory cells death and ameliorate disease progression. Currently, It has been found that anti-inflammatory cyclic dipeptide His-pro and diphenyl diselenide can reduce protein nitrification and inhibit NLRP3 inflammasome activation by reducing NO and ROS levels, thus playing a neuroprotective role in ALS model [125, 126].

####  HIV associated neurocognitive disorders

Combination antiretroviral therapy is the main anti-HIV treatment at present. Nearly 50% of infected people in the world have received cART. Although cART is able to prolong survival. Nearly 50% of those infected continue to suffer from human immunodeficiency virus (HIV)-associated neurocognitive disorders (HANDs) such as learning, memory and executive dysfunction [127, 128]. Antiretroviral drug-related neurotoxicity, persistent low levels of viral replication in the CNS, viral proteins such as HIV-1envelope protein gp120 and proinflammatory cytokines are involved in HIV-1-mediated neurodegeneration [129–131]. Walsh et al. found ASC ectopia, caspase1 cleavage, and IL-1β release in human microglia exposed to HIV-1 gp120 increased. NLRP3 deficiency and elevated extracellular K^+^ inhibited HIV-1-mediated IL-1β release [132]. Similar to the aforementioned experimental result, Chivero et al. found that HIV-1 trans-activator of transcription protein can promote caspase1 cleavage and IL-1β release in rat glial cells, and blocking NLRP3 can reduce IL-1β secretion. This result was verified in brain tissue from SIV-infected monkeys [133]. Further studies have confirmed that gp120LAV could bind to CXCR4 of microglia after shedding from the virus surface during HIV-1 infection, thus promoting nuclear translocation of NF-κB P65 and K^+^ efflux. NF-κB activation could induce NLRP3priming, and K^+^ efflux could promote NLRP3 inflammasome assembly, pyroptosis and IL-1β release, ultimately leading to neuronal death. And MCC950 showed a good neuroprotective effect on gp120LAV-induced neuronal death and dysfunction [134]. Therefore, NLRP3 inflammasome activation may be a potential target for HIV/AIDS therapeutic intervention, and NLRP3 inhibitors, IL-1 and other downstream inflammatory cytokine receptor antagonists hold the promise of being therapeutic drugs to improve HANDs.

###  X-linked adrenoleukodystrophy

X-linked adrenoleukodystrophy (X-ALD) is a hereditary metabolic disease composed of the accumulation of very long-chain fatty acids in adrenal cortex, CNS and plasma induced by ATP- binding cassette subfamily D member 1 (ABCD1) gene mutation. Its pathogenesis is not yet clear [135]. Jang et al. found increased expression of cholesterol 25-hydroxylase in fibroblasts of X-ALD patients with ABCD1 dysfunction and brain homogenates of ABCD1-deficient mice, and further confirmed that 25-HC could promote the NLRP3 inflammasome and caspase-1 activation and IL-1β production through potassium efflux, mtROS production, mitochondrial damage, and liver X receptor, thus inducing microglia recruitment and oligodendrocyte death [136]. Knockdown of NLRP3 attenuates 25-HC-mediated neuroinflammation and plays a neuroprotective role. Therefore, NLRP3 may be a potential therapeutic target for X-ALD.

###  Glioma of the CNS

Glioma is the most common and fatal primary CNS tumor in adults. Surgery, chemoradiotherapy and other regimens commonly used at present can not significantly improve patient prognosis, quality of life and survival time [137, 138]. It was found that NLRP3, ASC, caspase-1 and IL-1β were over-expressed in human glioma tissues and significantly correlated with the World Health Organization classification. Down-regulation of NLRP3 could reduce the expression of ASC, caspase-1 and IL-1β, inhibit epithelial-mesenchymal transition and PTEN/AKT signaling pathway, thus inhibiting the proliferation, migration and invasion of glioma cell lines [139]. Xue et al. found that knockdown of NLRP3 inhibited the growth and invasion of glioma cells, and also decreased the expression of IL-1β and NF-κB p65. Silencing NLRP3 inhibited the proliferation and invasion of tumor cells promoted by exogenous IL-1β.Eliminating IL-1β attenuated the pro-proliferative effects of NLRP3 overexpression. Blocking NF-κB inhibited the overexpression of IL-1β and NLRP3 [140]. These results indicate that NLRP3 can regulate the IL-1β/NF-κB p65 signaling pathway to promote the growth and invasion of glioma. To sum up, inhibiting NLRP3 inflammasome activity and its signaling pathway can inhibit the growth and invasion of glioma cells. Ding et al. found that miR-223-3p expression was decreased, while NLRP3 was increased in glioma tissues. MiR-223-3p mimics could inhibit the proliferation and migration of tumor cells by down-regulating the expression of various inflammatory cytokines such as IL-1β, monocyte chemoattractant protein-1, IL-8 and IL-18. And up-regulating NLRP3 expression could attenuate the effect of mir-223-3p mimics [141]. Role of mir-223-3p in negative regulation of NLRP3-mediated inflammatory response makes it likely to be an inhibitory factor of glioma, slow down the disease process and prolong the survival time of patients.β-hydroxybutyrate was reported to inhibit the activation of NLRP3 inflammasome and the expression of caspase-1 and IL-1β in C6 glioma cells, thus inhibiting the migration of C6 cells [142]. IP-Se-06, a selenylated imidazo [1,2-a] pyridine, was also found to reduce NLRP3 and caspase-1 levels in A172 glioblastoma cells [143].

###  Epilepsy

Epilepsy is a common end-point of many brain disorders such as tumor, infection, immune abnormality, metabolic disorder and traumatic brain injury. It can be the result of a single gene mutation (hereditary epilepsy) or a component of neurodevelopmental disorder [144]. Epilepsy affects approximately 1% of the population of all ages worldwide, refractory epilepsy accounts for more than 30%. Patients with epilepsy often require long-term and regular medication, but refractory epilepsy cannot be well controlled by existing drugs [145]. Therefore, it is urgent to develop new drugs to treat epilepsy. At present, the cellular and molecular mechanisms of epilepsy are still unclear, but a large amount of clinical and experimental evidence indicates that CNS or systemic inflammatory disorders and their mediated neuronal damage, gliosis cell proliferation, abnormal neural connections and hyperexcitable neural networks play an important role in the development of epilepsy [146, 147]. Meng et al. demonstrated for the first time that NLRP3 and IL-1β levels increase after status epilepticus in rat hippocampus, blocking NLRP3 inflammasome assembly or Caspase-1 activation could reduce the serum IL-1β and IL-18 levels, the number and severity of seizures after SE, and slow down the disease progression. knocking out NLRP3 or caspase-1 at 6 weeks after SE could maintain neuronal activity and reduce neuronal loss in hippocampal CA1 and CA3 regions [148]. Wu et al. found that the NLRP3 level in the epileptogenic brain tissue of the children with intractable temporal lobe epilepsy was higher than that in the normal brain tissue adjacent to the epileptogenic zone, and the IL-1β level in peripheral blood was higher than that in healthy children, which was positively correlated with the onset time of single seizure [149]. Qin et al. demonstrated that GPR120 overexpression reduced neuronal death after SE through down-regulating NLRP3 inflammasome expression, and knocking down GPR120 showed the opposite effect [150]. Therefore, inhibiting the activity of NLRP3 inflammasome and its downstream inflammatory cytokines is essential to reduce the occurrence of epilepsy and control its progression. Rong et al. found that amentoflavone could inhibit pentylenetetrazol-induced NLRP3 inflammasome activation and the release of IL-18, IL-1β and TNF-α in mice hippocampus, thus reducing seizures and playing a neuroprotective role [151]. He et al. demonstrated that curcumin could inhibit NLPR3 inflammasome activation and reduce hippocampal neuron loss during epilepsy [152]. Moreover, ibuprofen, CY-09, MCC950, neferine, eicosapentaenoic acid and docosahexaenoic acid have been reported to inhibit NLRP3 inflammasome activation to reduce neuroinflammation, exert anti-epileptic and neuroprotective effects [153–157]. Traditional Chinese medicine such as Chaihu-Longgu-Muli decoction was also found to exert anti-epileptic effect by inhibiting nlrp3 activity [158].

##  The applied perspectives of NLRP3 inhibitors

To sum up, inhibition of NLRP3 inflammasome signaling pathway has shown good protective effect in a variety of animal models of CNS disease. NLRP3 is a potential therapeutic target for CNS disease. The development and clinical application of NLRP3 inflammasome and its downstream inflammatory cytokine inhibitors are of great value. Unfortunately, several inhibitors has become available show adverse reactions in clinical trials. The phase II clinical trials of MCC950 (specific NLRP3 inflammasome inhibitor) in the treatment of rheumatoid arthritis was suspended due to liver toxicity. The furan structure of MCC950 is considered to be one of the causes of drug-induced liver damage [159]. GDC-2394 is an oral selective NLRP3 inhibitor, which was first published in 2022. Because it removed furan structure extensively compared with MCC950, introduced basic amine into the scaffold to increase solubility and reduce nephrotoxicity, and successfully passed the safety study of cynomolgus monkeys. So researchers place high hopes that it will be able to complete clinical trials [160]. However, in a phase 1 clinical trial that evaluated the safety, pharmacokinetics and pharmacodynamics of GDC-2394 in healthy volunteers, two volunteers developed grade 4 drug-induced liver damage during the GDC-2394-midazolam interaction phase, so the trial was suspended. Indoleamine, a metabolite of GDC-2394, is considered to be associated with hepatotoxicity, and the concentration of indoleamine after GDC-2394 alone is the same as that after GDC-2394 combined with midazolam, so midazolam is not considered to be the perpetrator of liver damage [161]. However, this does not mean that NLRP3 inhibitors have no future. At present, more and more new inhibitors have been developed or gradually passed clinical trials.Elnoflast (RO7486967) is a reversible small molecule selective NLRP3 inhibitor. A randomized, double-blind phase 1b clinical trial confirmed that Selnoflast at a dose of 450 mg/day for 7 days was well tolerated without serious adverse reactions, and successfully reached the expected level of plasma and tissue exposure [162]. DFV890 is an oral selective NLRP3 inhibitor that can inhibit the activity of NLRP3 by directly binding to NLRP3 and locking it in an inactive conformation. A human safety, tolerance and pharmacokinetic experiment showed that 100 mg once a day or 25 mg twice a day of DFV890 was sufficient to stably inhibit 90% of IL-1 β release.10% of the subjects developed mild to moderate intensity of papules and / or itching rash at the dose of 100 mg QD or 30 mg bid.And the rash subsided spontaneously after discontinuation, without involving the epidermis, and no serious adverse reactions occurred. However, no subjects developed a rash at the dose of 25 mg bid, suggesting that the low dose DFV890 was well tolerated by healthy subjects and had no safety problems [163]. Dapansutrile (OLT1177) is an oral small molecule selective NLRP3 inhibitor, which can directly target NLRP3 by inhibiting ATPase activity. In the phase 1 study of OLT1177 in healthy subjects, no serious adverse reactions occurred, showing good safety and tolerance [164]. In a phase 2a clinical trial of gout patients, low\medium and high doses of OLT1177 significantly reduced the joint swelling and pain of the subjects, and almost no adverse events related to OLT1177 occurred [165]. In the phase 1B safety and pharmacodynamics study of NYHAII-III systolic heart failure patients, OLT1177 at a dose of 2000 mg/ day increased subjects’ the left ventricular ejection fraction from 31.5% (27.5–39) to 36.5% (27.5–45) (*P* = 0.039). And there were no drug-related water and sodium retention, renal function damage, heart rate and blood pressure abnormalities during the treatment [166]. In a word, the prospect of developing and applying NLRP3 inhibitors to treat CNS diseases is still bright.

## Conclusion

NLRP3 inflammasome has long been a research hotspot, which has been proved to play an important role in the occurrence and development of infectious, inflammatory immune, vascular, genetic metabolic, tumor, epilepsy and other diseases in the CNS. Although there is mounting evidence that a variety of NLRP3 inhibitors show good efficacy in preclinical immunopathological models such as AD, TBI, atherosclerosis, diabetic encephalopathy, HANDs, cerebral edema, cerebral ischemia-reperfusion injury and ischemic hypoxic encephalopathy [1–6]. But the activation of inflammasome is crucial to the defense of pathogens, and the loss of IL-1β and other inflammatory cytokines may adversely affect the immune defense of the body, only specific NLRP3 inflammasome inhibitors have a chance of being used clinically. The side effects of MCC950 and others in clinical trials cast a shadow on its clinical application, but OLT1177 and others have successively completed phase 1 clinical trials, which makes us firmly believe that the prospect of targeting NLRP3 inflammasome in the treatment of CNS diseases is bright.With the deepening of research and the rapid development of molecular biology, the molecular principles of inflammasome assembly will be uncovered. NLRP3 is expected to be a potential target for the treatment of CNS diseases, and will play a greater role in the treatment of CNS diseases.

## Data Availability

The datasets used and/or analysed during the current study are available from the corresponding author on reasonable request.

## Abbreviations

CNS: central nervous system
PYD: pyrin domain
LRR domain: leucine-rich-repeats domain
HIV: human immunodeficiency virus
HANDs: HIV-associated neurocognitive disorders
NF: κB: factor-κB
POPs: pyrin-only proteins
COPs: card-only proteins
ZIKV: Zika virus
EV71: enterovirus 71
S. pneumoniae: Streptococcus pneumoniae
TBI: traumatic brain injury
NMDAR: Anti-N-methyl-d-aspartate receptor
GFAP: autoimmune glial fibers acidic protein
NMOSDs: neuromyelitis optica spectrum disorders
MS: multiple sclerosis
AD: alzheimer’s disease
Aβ: amyloid-β
PD: parkinson’s disease
ALS: amyotrophic lateral sclerosis
SOD1: superoxide dismutase
X-ALD: X-linked adrenoleukodystrophy
ABCD1: ATP- binding cassette subfamily D member 1
ICH: Intracerebral hemorrhage
SAH: Subarachnoid hemorrhage
